# Investigating the Genetic Profile of the Amyotrophic Lateral Sclerosis/Frontotemporal Dementia (ALS-FTD) Continuum in Patients of Diverse Race, Ethnicity and Ancestry

**DOI:** 10.3390/genes13010076

**Published:** 2021-12-28

**Authors:** Maysen Mesaros, Steven Lenz, Woobeen Lim, Jordan Brown, Luke Drury, Jennifer Roggenbuck

**Affiliations:** 1 Department of Internal Medicine, The Ohio State University Wexner Medical Center, Columbus, OH 43212, USA; Jordan.brown@osumc.edu (J.B.); jennifer.roggenbuck@osumc.edu (J.R.); 2 Division of Neuroscience, Medical University of South Carolina, Charleston, SC 29425, USA; 3 PreventionGenetics, Marshfield, WI 54449, USA; steven.lenz@preventiongenetics.com (S.L.); luke.drury@preventiongenetics.com (L.D.); 4 Division of Biostatistics, College of Public Health, The Ohio State University, Columbus, OH 43210, USA; lim.1148@osu.edu

**Keywords:** ALS, FTD, REA, genetics, *C9orf72*

## Abstract

Preliminary evidence suggests that commonly used genetic tests may be less likely to identify a genetic etiology for ALS-FTD in patients of underrepresented race, ethnicity, and ancestry (REA), as compared to European REA. Patients of underrepresented REA may therefore be less likely to receive accurate and specific genetic counseling information and less likely to have access to gene-targeted therapies currently in clinical trials. We compiled outcome data from 1911 ALS-FTD patients tested at a commercial laboratory over a seven-year period for *C9orf72* hexanucleotide repeat expansion (HRE) alone or *C9orf72* and multigene sequencing panel testing. We compared the incidence of pathogenic (P), likely pathogenic (LP), and uncertain variants in *C9orf72* and other ALS-FTD genes, as well as age at testing, in patients of different REA. The diagnostic rate in patients of European REA (377/1595, 23.64%) was significantly higher than in patients of underrepresented REA (44/316, 13.92%) (*p* < 0.001). Patients of European REA were more likely to have the *C9orf72* HRE (21.3%) than patients of underrepresented REA (10.4%) (*p* < 0.001). The overall distribution of positive test outcomes in all tested genes was significantly different between the two groups, with relatively more P and LP variants in genes other than *C9orf72* identified in patients of underrepresented REA. The incidence of uncertain test outcomes was not significantly different between patients of European and underrepresented REA. Patients with positive test outcomes were more likely to be younger than those with negative or uncertain outcomes. Although *C9orf72* HRE assay has been advocated as the first, and in some cases, only genetic test offered to patients with ALS-FTD in the clinical setting, this practice may result in the reduced ascertainment of genetic ALS-FTD in patients of diverse REA.

## 1. Introduction

Amyotrophic lateral sclerosis (ALS) is an adult-onset neurodegenerative disease characterized by the loss of upper and lower motor neurons. Individuals with ALS experience progressive paralysis that ultimately results in death within an average of three to five years after symptom onset [1]. ALS has long been recognized to form a neurodegenerative continuum with frontotemporal dementia (FTD), a progressive disorder affecting behavior and language, by a common underlying TDP43 neuropathology [2]. It has been reported that up to 50% of individuals with ALS develop cognitive impairment associated with FTD, and up to 30% of individuals with FTD develop symptoms of motor neuron disease [3]. The recent discoveries of common genetic etiologies have led to a growing recognition that ALS and FTD represent opposite ends of one common phenotypic spectrum [2,4]. 

ALS affects individuals of all races, ethnicities, and ancestries (REA); however, the incidence rates vary across REA groups in the United States of America. The European or white REA group has the highest incidence, followed by African American or black, then Asian. Non-Hispanic REA has a higher incidence of ALS than Hispanic REA [5]. The incidence of FTD is also shown to vary across populations; however, data are primarily based on the study of Caucasian patients, and evidence regarding the incidence of FTD in diverse populations is particularly limited [6]. While differences in incidence rates may reflect multiple factors, such as genetic predisposition, environmental exposures, access to adequate healthcare, or lack of ascertainment of underrepresented REA cases in incidence studies, evidence suggests that these variations in incidence are less likely to result from ascertainment bias and socioeconomic status and more likely to result from genetic variation across REA groups [7].

In addition to the variation in incidence rates of ALS across REA groups, clinical presentations of the disease differ across groups. The average age of onset of ALS symptoms is younger in Black or African American people (50–59 years) than that of individuals in other REA groups (60–69 years). Hispanics have an average age of onset that is approximately four years lower than the age of onset for non-Hispanic patients. The mean duration from symptom onset to diagnosis is lowest among African Americans (15.6 months) and highest among Asians (20.4 months) [5]. A meta-analysis of ALS outcomes across 40 geographical regions of the world demonstrated that the median survival time was lowest among those in Northern European cohorts (25 months). The median survival time in Asia ranged from 28 months in East Asia to 48 months in Iran. Data on the median survival in African or African-American cohorts is limited; however, some studies in the United States of America demonstrated that patients of African origin had similar survival times to those of European origin [8], while a 2019 study in a cohort of 49 African American patients with ALS found longer survival times and higher rates of tracheostomy and invasive ventilation than in white patients [9]. The diversity in presentation of ALS across REA groups could reflect diversity in genetic etiologies underlying these differences. Differences in disease presentation of FTD across REA groups have not been well studied [2].

A genetic etiology is identified in approximately 70% of familial ALS (fALS) and familial FTD (fFTD) cases, 15% of sporadic ALS (sALS) cases, and 7% of sporadic FTD (sFTD) in North American research cohorts, which consist primarily of patients of European descent [2,10,11,12]. Genetic forms of the ALS and FTD spectrum, hereafter designated ALS-FTD, are typically inherited in an autosomal dominant manner with significant genetic heterogeneity. Pathogenic variants in > 40 genes have been identified to cause either ALS, FTD, or ALS-FTD [12,13]. Genes recognized as causative for both ALS and FTD include *C9orf72, TARDBP, SQSTM1, VCP, FUS, TBK1, CHCHD10*, and *UBQLN2* [2]. The pathogenic hexanucleotide repeat expansion (HRE) in the *C9orf72* gene is the most identified genetic cause of both ALS and FTD. This gene was discovered in 2011 and is responsible for approximately 40% of fALS and fFTD cases, and up to 7% of sALS and sFTD cases [2,12]. 

Genetic and historical evidence suggests that the *C9orf72* HRE first appeared in Scandinavia approximately 1500 years ago and spread throughout Europe with the conquests of Vikings. The geographical distribution of the *C9orf72* HRE and Viking routes are remarkably similar, supporting this theory on the spread of this variant [14,15]. Today, it is believed that the *C9orf72* HRE has remained most prevalent in European patients. In a 2012 study of a global cohort of 4448 individuals with ALS and 1425 with FTD, the frequency of *C9orf72* HREs was determined across different ethnic groups; clear variation was found with the highest frequency in patients of European REA [16,17,18]. Distributions of pathogenic variants in other genes causing ALS-FTD are also believed to be uneven across populations [17,19,20,21,22,23,24,25,26]. However, data comparing the prevalence of genetic variants in ALS-FTD across REA groups is limited, and the impact of this variation on the diagnostic yield of clinical genetic testing has not been studied. 

Current US ALS management guidelines do not address the offer of genetic testing, and there is a lack of consensus among clinicians on what testing should be offered to patients and in what clinical scenarios [27,28]. Commercially available genetic tests for ALS-FTD include single-gene sequencing, multigene sequencing panels, the *C9orf72* HRE assay, and whole-exome sequencing [12]. Since the *C9orf72* HRE cannot be detected via sequencing, clinicians must order the HRE assay separately. Some clinics offer testing for the *C9orf72* HRE as the first and sometimes only test for patients with ALS-FTD due to its high prevalence in patients of European REA [11,29,30]. The impact of this approach on genetic diagnosis in patients of underrepresented REA is unknown; however, we suspect that this practice may result in a reduced ascertainment of genetic ALS-FTD in patients of underrepresented REA. 

Missed genetic diagnoses are expected to negatively impact the care of patients of underrepresented REA. The current understanding of gene-disease relationships is based primarily on the study of individuals of European REA, and patients of underrepresented REA are less likely than Europeans to receive a definitive genetic diagnosis for their disease across many medical specialties [31,32,33,34,35,36]. As a result, the current effort to employ precision medicine to drive the treatment of hereditary disease threatens to disadvantage patients of diverse REA. It is crucial that efforts are made to improve access to genetic diagnosis to ensure access to accurate and specific genetic counseling information, participation in gene-specific natural history studies, and eligibility for gene-targeted interventional trials for all patients. In order to investigate the diagnostic yield of ALS-FTD genetic testing in patients of different REA, we examined the test outcomes in a large commercial laboratory cohort.

## 2. Materials and Methods

**Figure 1:**
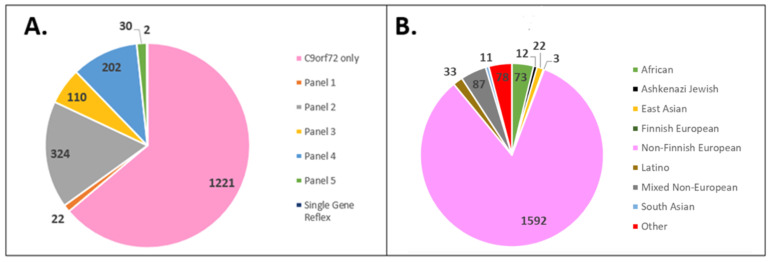
(**A**) Distribution of tests ordered (panel genes listed in Table 1). (**B**) Cohort REA distribution.

This study was deemed exempt from review by The Ohio State University Institutional Review Board (IRB) in Columbus, Ohio. A retrospective review of a commercial laboratory testing database was performed to identify a cohort dataset consisting of all patients who underwent ALS-FTD genetic testing at the Prevention Genetics Laboratory from 2013 to 2020. The cohort included representation from both the USA and Canada with samples received from multiple different national ALS-FTD centers and Canadian provinces. This dataset included 1911 individuals of diverse REA between the ages of 8 and 96 years old who underwent *C9orf72* HRE analysis exclusively or *C9orf72* HRE analysis with reflex to multigene-panel or single-gene analysis (Table 1, Figure 1A). *C9orf72* repeat expansions were analyzed using two custom repeat primed PCR assays for both the 3′ and 5′ ends of the HRE. *C9orf72* repeats of ≥ 30 were considered pathogenic, 25–29 considered intermediate and of uncertain clinical significance, and < 25 repeats normal. For patients tested via panel testing, *C9orf72* repeat expansion testing was performed first, and if negative, reflexive panel testing via next-generation sequencing was conducted. Sequencing was performed using Illumina NovaSeq 6000 (Illumina, San Diego, CA, USA) and reads were aligned to a reference sequencing (hg19). All variants were interpreted per the American College of Medical Genetics variant classification guidelines, and only uncertain, likely pathogenic, and pathogenic variants were reported [37]. 

The collected data included the clinician-reported patient ancestry, patient year of birth, test ordered, date of test order, and test result including variant(s) identified and interpretation. All tested individuals with REA information provided were sorted into nine REA groups, closely matching ancestral groups in the gnomAD population database (African/African American, Latino, Ashkenazi Jewish, East Asian, European (Finnish), European (Non-Finnish), Other, South Asian, and Mixed Non-European). Genetic test outcomes and ages at testing were then compared between REA groups. Cases were collapsed into European (European (Finnish), European (Non-Finnish)) and Underrepresented REA (African/African American, Ashkenazi Jewish, Latino, East Asian, Other, South Asian, Mixed Non-European) for most analyses. Analysis was performed for cases that underwent *C9orf72* HRE testing exclusively and for those that had *C9orf72* HRE testing followed by multigene or single gene sequencing. For cases that underwent *C9orf72* HRE testing, Fisher’s exact test was performed to determine the distribution of positive *C9orf72* HREs across REA groups. For tests performed on the distribution of multigene panel results, distribution of testing ordered, and cohort diagnostic yields, chi-square testing was performed to identify significant differences across REA groups. One-way ANOVA tests were performed to determine if age at testing differed by genetic test outcome. A two-way ANOVA test was performed to determine if the age at testing differed by the genetic test outcome between those of European and underrepresented REA.

## 3. Results

### 3.1. Cohort Demographics 

Among 1911 cases in the tested cohort, 1592 (83.3%) were classified as European Non-Finnish, 73 (3.82%) as African/African American, 12 (0.63%) as Ashkenazi Jewish, 22 (1.15%) as East Asian, 3 (0.16%) as European Finnish, 33 (1.73%) as Latino, 87 (4.55%) as Mixed Non-European, 11 (0.58%) as South Asian, and 78 (4.08%) as other (Figure 1B). After the European and underrepresented REA groups were collapsed, 1595 (83.46%) of the cohort fell into the European REA with an average age at testing of 61.12 years, and 316 (16.54%) of the cohort fell into underrepresented REA with an average age at testing of 57.99 years. 

### 3.2. Positive Test Outcomes across REA Groups

**Figure 2:**
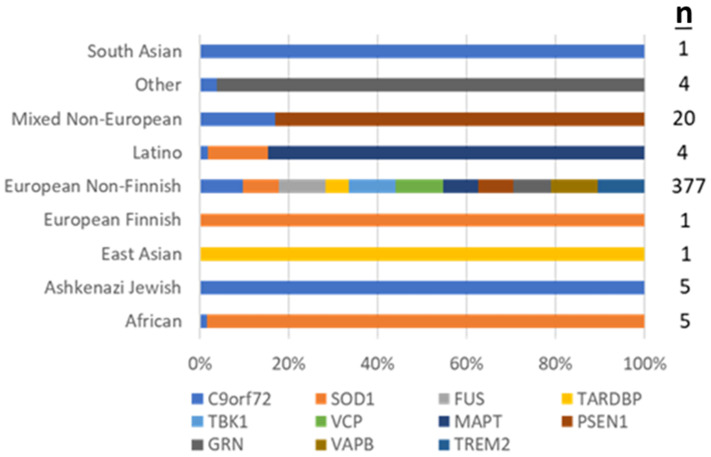
Pathogenic/likely pathogenic variants identified, by patient REA.

The overall diagnostic yield of genetic testing in the cohort (pathogenic (P) or likely pathogenic (LP) variant identified in an autosomal dominant gene or two P or LP variants in a recessive gene) was 421/1911 (22.03%). Testing was more likely to establish a genetic diagnosis in patients of European REA (377/1,595, 23.64%) than for patients of underrepresented REA (44/316, 13.92%) (*p* < 0.001). Among patients of African/African American REA, 5/73 (6.85%) were found to have the *C9orf72* HRE and 4/73 (5.48%) had P/LP variants in *SOD1*. Among patients of Ashkenazi Jewish REA, 5/12 (41.67%) were found to have the *C9orf72* HRE. Among patients of East Asian REA, 1/22 (4.55%) had a P/LP variant in *TARDBP*. Among patients of European Finnish REA, 1/3 (33.33%) were found to have an LP in *SOD1*. Among patients of European Non-Finnish REA, 339/1592 (21.29%) were found to have the *C9orf72* HRE, 19/1592 (1.19%) had P/LP variants in *SOD1*, 3/1592 (0.19%) had P/LP variants in *FUS*, 3/1592 (0.19%) in *MAPT*, and 2/1592 (0.13%) had variants in *TBK1*; a P/LP variant in *TARDBP*, *VCP*, *TREM2*, *VAPB*, *GRN*, *PSEN1* was identified in one case each. Among patients of Latino REA, 2/33 (6.06%) had the *C9orf72* HRE, and a P/LP variant in *SOD1* and *MAPT* were found in one case each. Among patients of Mixed Non-European REA, 17/87 (19.54%) were found to have the *C9orf72* HRE and 1/87 (1.15%) had a P variant in *PSEN1*. Among patients of Other REA, 3/78 (3.85%) had the *C9orf72* HRE and 1/39 (2.56%) had a LP variant in *GRN*. Among patients of South Asian REA, 1/11 (9.09%) tested positive for the *C9orf72* HRE (Figure 2). All variants detected by panel sequencing and case clinical details are provided in the Supplementary Data section (Table S1).

The incidence of the *C9orf72* HRE was significantly different across individual REA groups (*p* < 0.001, Table 2) and between the European and underrepresented REAs (*p* < 0.001, Table 3). Patients of European REA were more likely to test positive for the HRE than patients of underrepresented REA (*p* < 0.001). 

The overall rate of positive, negative, and uncertain results on sequencing panel testing was determined across all REA groups and between European and underrepresented patients who underwent panel testing (see Table 1 for panels conducted). The overall distribution of these test outcomes was found to be different across all REA groups (*p* = 0.012, Table 4), with the highest positive yield in patients of African/African American REA, (16.7%) and lowest in patients identified as Ashkenazi Jewish or Asian (0%). When REA groups were collapsed to European and underrepresented and compared, the overall proportion of positive, negative, and uncertain results was not significantly different (*p* = 0.64), suggesting that there are differences in the outcomes of panel testing between individual REA groups, such as between African/African Americans and Asians. 

The overall distribution of positive results was compared between patients of European and underrepresented REA. Patients of underrepresented REA were more likely to test positive for sequencing panel genes (i.e., genes other than *C9orf72*) compared to patients of European REA (*p* = 0.007, Table 5). The *C9orf72* HRE accounted for 90% of positives identified in the European REA group and 75% of positives in the underrepresented REA group. Pathogenic and likely pathogenic variants in genes other than *C9orf72* accounted for 10% of positives in the European REA group, while they accounted for 25% in the underrepresented REA group.

### 3.3. Uncertain Result Outcomes across REA Groups

The proportion of uncertain results did not significantly differ by REA group for those that underwent sequencing panel testing. This was observed comparing all REA groups individually (*p* = 0.142) and after collapsing European and underrepresented REA (*p* = 0.459). The highest rate of uncertain results was found in patients identified as “Other” REA (26.3%), while the lowest rate was found in patients identified as Ashkenazi Jewish (0.0%). The rate of uncertain test outcomes was 8.9% and 11.6% for patients of European and underrepresented REA, respectively.

### 3.4. Age at Testing across REA Groups

The patient age at testing and test outcomes were compared across the cohort and across REA groups. For patients who underwent *C9orf72* HRE testing, those with positive results were more likely to be younger than those with negative or intermediate results (*p* = 0.006, Table 6). Patients who had positive results on panel testing also tended to be younger than those with negative or uncertain results, though this did not reach statistical significance (*p* = 0.063, Table 7). Although younger patients were more likely to test positive regardless of REA, those who tested positive of underrepresented REA were younger than those of European REA (*p* < 0.001).

### 3.5. Testing Ordered across REA Groups

The type of test ordered (*C9orf72* HRE analysis exclusively versus the addition of multigene panel testing) did not differ between patients with European vs. underrepresented REA (*p* = 0.87). 

## 4. Discussion

The results of this study appear to confirm that genetic testing for ALS-FTD, as currently practiced, is less likely to establish a genetic diagnosis in patients of underrepresented REA compared to those of European REA. However, it is not clear if this is due to a higher incidence of genetic ALS-FTD in patients of European REA [5,6], or a reduced ascertainment of genetic ALS-FTD in other populations, or, as we suspect, both. While some studies have reported that white patients with ALS are more likely to have a family history of ALS than African American/Black, Asian, and Hispanic individuals [5], perhaps suggesting a higher likelihood of a genetic etiology, patients of underrepresented REA may be less likely to have complete family history information [38,39,40]. Additionally, in one study of an ALS register in the southern USA, the rate of familial cases was not significantly different between African American and Caucasian cases [41]. Further study of ALS-FTD and its genetic basis in populations of underrepresented REA is necessary to address this question. 

This study further confirmed that the *C9orf72* HRE is the most common pathogenic variant identified in patients with ALS-FTD in clinical practice in North America, both in patients of European and underrepresented REA. While the HRE was identified in ~10% of the patients of underrepresented REA overall, the REA-specific rate ranged from 0% in Asian to 6.8% in African/African American to 41.7% in Ashkenazi Jewish. Although our REA-specific data are limited due to small numbers in the gnomAD categories, these observations appear to be in line with the published HRE frequency data. This cohort’s relatively high frequency of HRE in Ashkenazi Jewish patients matches that of a previous report on an Israeli study population wherein 80% of fALS cases and 11% of sALS cases were found to carry the HRE [42]. The HRE has been previously reported at frequencies of 3.13% in Black patients with ALS, 0% in Middle Eastern patients with sALS and fALS, 0% in Asians with sALS, 0% in Asians with sFTD, and 5% in Asians with fALS [16,41]. 

Notably, a significantly higher proportion of patients of underrepresented REA tested positive in genes other than *C9orf72* compared to patients of European REA. Patient REA (European vs. underrepresented) did not appear to be associated with the type of testing ordered (*C9orf72* HRE analysis exclusively versus the addition of multigene panel testing) (*p* = 0.87). This suggests that clinicians are not considering patient REA in their ordering practices. Our results may reflect clinician bias towards offering the *C9orf72* HRE as a sole test; had more patients of underrepresented REA had more comprehensive testing, the proportion of positives in genes other than *C9orf72* could potentially be even higher. It is unclear from this data why clinicians ordered *C9orf72* HRE analysis exclusively in some cases while in others ordered the analysis of additional genes. Although this could reflect clinical factors not included in our dataset, such as family history, it could also reflect variable and inconsistent clinical practice. Genetic testing guidelines for ALS-FTD are needed to improve the diagnosis in underrepresented and all ALS-FTD patients worldwide.

Our data suggest that the offer of the *C9orf72* HRE analysis as a first test to patients of underrepresented REA is indicated in clinical practice in North America and potentially other geographic areas with a history of European colonization and admixture across REA groups. However, additional genetic testing should be offered for patients of underrepresented REA who test negative for the HRE, given that a higher proportion are expected to have pathogenic variants in other genes, such as *SOD1*. The offer of *C9orf72* HRE assay as a sole genetic test may result in reduced ascertainment of genetic ALS-FTD in patients of underrepresented REA and is not recommended for this group. 

In this large cohort, the rate of uncertain test outcomes (results with variant(s) of uncertain significance as the only finding) was not significantly different between patients of European and underrepresented REA. Similarly, a study of genetic test outcomes in a midwestern US ALS clinic reported that uncertain test outcomes were not more common in non-Caucasian than Caucasian patients [43]. This finding contrasts with genetic test outcome data from other disease groups including cancer and cardiology, wherein higher rates of uncertain results are observed in patients of underrepresented REA [33,34,44]. Such findings are often attributed to the lack of adequate representation of diverse REA groups in population databases [31,45]. Nonetheless, many patients in the tested cohort had uncertain test outcomes, ranging from 4.2% to 26.3% in different REA groups. In ALS-FTD, options for investigation of specific variants, such as functional studies or segregation analysis, may be limited [43]. 

These test outcome data reveal that patients with positive test results were more likely to be younger than patients with negative or uncertain results. Additionally, of those that tested positive, patients of underrepresented REA were more likely to be younger than those of European REA. This could reflect increased exposure to environmental or lifestyle risk factors, or genetic factors. We found that patients of underrepresented REA were relatively more likely to have pathogenic variants in genes other than *C9orf72* compared to Europeans, and such genes (including *SOD1*) have been associated with earlier presentations of ALS symptoms than *C9orf72* [46]. This finding may suggest that patients with an early onset of symptoms are more likely to have an identifiable genetic etiology, particularly those of underrepresented REA. While the European Federation of Neurological Societies (EFNS) recommends the offer of genetic testing for patients with familial ALS, there is no specific recommendation to test patients with an early onset of symptoms [47]. We suggest that the early onset of symptoms be considered as an additional, alternative criterion for the offer of genetic testing, particularly for patients of underrepresented REA who may be less likely to meet family-history-based testing criteria due to historically limited access to complete family history information [48,49]. We note, however, that early-onset ALS-FTD is poorly defined and should be specified if age of onset becomes a consideration in future genetic testing guidelines and protocols [12,50].

### Study Limitations

Although the aim of this study was to investigate the genetic profile of ALS-FTD in patients of diverse REA, patients of European REA comprised the majority of the studied cohort. Many analyses were limited by small numbers in individual REA groups, necessitating the collapsing of different groups into one underrepresented REA category, potentially obscuring important differences. In order to better investigate the genetic profile of specific REA groups, patients from those groups (i.e., African Americans) should be specifically recruited and studied. 

Another limitation to this study was that clinician-reported REA was used for this analysis as opposed to genetically determined ancestral assignment. Clinician-reported REA included a variety of geoancestry descriptors that were used as a proxy for the geoancestry categories used in the gnomAD database. The lack of consistent terminology and the intermixing of race, ethnicity, and ancestry terms when reporting REA information has been a barrier to investigating the impact of REA in clinical genetic testing [32,51]. As approximated REA was used for this study, the results of this study may be less accurate than if genetically determined ancestry was used for analysis. This limitation highlights the need for consistency in reporting and using REA information in genetic studies. 

## 5. Conclusions

As genetic testing becomes more widely adapted in patient care, it is crucial to understand the genetic profile of ALS-FTD across all REA groups. This study of a large North American clinical laboratory cohort identified significant differences in the genetic etiologies of ALS-FTD between REA groups and confirmed that genetic testing for ALS-FTD is more likely to establish a diagnosis in patients of European REA, compared to underrepresented REA. Our findings have several implications for clinical genetic testing practice: (1) the offer of the *C9orf72* HRE test is indicated for ALS-FTD patients of underrepresented REA in North America; (2) Additional comprehensive genetic testing should be offered for patients of underrepresented REA, either concurrently or as a second step if negative for the HRE; (3) patients with early-onset ALS-FTD should also be considered candidates for comprehensive genetic testing. 

Genetic testing protocols which maximize the identification of genetic ALS-FTD in patients of all REA are critically needed as gene-targeted therapies reach clinical trials. The development, evaluation, and publication of REA-specific or comprehensive pan-ethnic genetic testing guidelines will help all patients benefit from the coming age of personalized genetic medicine in ALS-FTD. 

## Figures and Tables

**Table 1:** Multigene sequencing panels performed in the tested cohort.

Panel 1 (28 gene ALS/dementia panel): *ALS2, ANG, ATXN1, CHCHD10, CHMP2B, DCTN1, ERBB4, FIG4, FUS, HNRNPA1, KIF5A, MATR3, NEFH, NEK1, OPTN, PFN1, PRPH, SETX, SIGMAR1, SOD1, SPG11, SQSTM1, TARDBP, TBK1, TUBA4A, UBQLN2, VAPB, VCP*
Panel 2 (24 gene ALS/dementia panel): *ANG, ANXA11, ARHGEF28, CDH13, CHMP2B, FUS, GRN, HNRNPA1, HNRNPA2B1, KIF5A, MAPT, OPTN, PFN1, PSEN1, PSEN2, SETX, SOD1, SQSTM1, TARDBP, TBK1, TREM2, UBQLN2, VAPB, VCP*
Panel 3 (11 gene dementia panel): *APP, CHMP2B, FUS, GRN, MAPT, PSEN1, PSEN2, SQSTM1, TARDBP, TREM2, UBQLN2*
Panel 4 (5 gene ALS panel): *FUS, SOD1, TARDBP, TBK1, VCP*
Panel 5 (3 gene ALS panel): *FUS, SOD1, TARDBP*
Single Gene Reflexes: *SOD1 and FIG4*

**Table 2:** Identification of the *C9orf72* HRE across all REA.

	REA									
	African/ African American (*n* = 73)	Ashkenazi Jewish (*n* = 12)	East Asian (*n* = 22)	European Finnish (*n* = 3)	European Non-Finnish (*n* = 1592)	Latino (*n* = 33)	Mixed Non-European (*n* = 87)	South Asian (*n* = 11)	Other (*n* = 78)	*p*-Value
Positive	5 (6.8%)	5 (41.7%)	0 (0.0%)	0 (0.0%)	339 (21.3%)	2 (6.1%)	17 (19.5%)	1 (9.1%)	3 (3.8%)	**<0.001**
Negative	68 (93.2%)	7 (58.3%)	22 (100.0%)	3 (100.0%)	1246 (78.3%)	31 (93.9%)	68 (78.2%)	10 (90.9%)	75 (96.2%)	
Intermediate	0 (0.0%)	0 (0.0%)	0 (0.0%)	0 (0.0%)	7 (0.4%)	0 (0.0%)	2 (2.3%)	0 (0.0%)	0 (0.0%)	

**Table 3:** Identification of the *C9orf72* HRE in European and underrepresented REA.

	REA Group		*p*-Value
Result	European (*n* = 1595)	Underrepresented (*n* = 316)	**<0.001**
Positive	339 (21.3%)	33 (10.4%)	
Negative	1249 (78.3%)	281 (88.9%)	
Intermediate	7 (0.4%)	2 (0.6%)	

**Table 4:** Identification of positive, negative, and uncertain results on multigene sequencing panels across all REA groups.

	REA									
Results (*n*, %)	African/ African American (*n* = 24)	Ashkenazi Jewish (*n* = 5)	East Asian (*n* = 10)	European Finnish (*n* = 1)	European Non-Finnish (*n* = 575)	Latino (*n* = 20)	Mixed Non-European (*n* = 26)	South Asian (*n* = 8)	Other (*n* = 19)	*p*-Value
Positive	4 (16.7)	0 (0.0)	0 (0.0)	1 (100.0)	38 (6.6)	2 (10.0)	1 (3.8)	0 (0.0)	1 (5.3)	**0.012**
Negative	19 (79.2)	5 (100.0)	8 (80.0)	0 (0.0)	486 (84.5)	17 (85.0)	23 (88.5)	6 (75.0)	13 (68.4)	
Variant of Uncertain Significance	1 (4.2)	0 (0.0)	2 (20.0)	0 (0.0)	51 (8.9)	1 (5.0)	2 (7.7)	2 (25.0)	5 (26.3)	

**Table 5:** Distribution of positive results of all ALS genes across mixed REA.

Test (*n*, %)	European (*n* = 377)	Underrepresented (*n* = 44)	*p*-Value
C9 Positive	339 (90)	33 (75)	**0.007**
Multigene Panel Positive	38 (10)	11 (25)	

**Table 6:** Distribution of age at testing across *C9orf72* HRE test outcomes.

	Positive	Intermediate	Negative	*p*-Value
*n*	372	9	1530	**0.006**
Age at testing (Mean, SD)	58.76 (11.54)	59.56 (14.29)	59.56 (14.29)	

**Table 7:** Distribution of age at testing across gene panel outcomes.

	Positive	Non-Positive	*p*-Value
*n*	47	641	**0.063**
Age at testing (Mean, SD)	57.26 (12.70)	60.78 (12.35)	

## Supplementary Materials

The following are available online at https://www.mdpi.com/article/10.3390/genes13010076/s1: Table S1: Variants Detected by Gene Panel Testing on *C9orf72* Negative Patients.
