# Supplementary material for: Investigating the Genetic Profile of the Amyotrophic Lateral Sclerosis/Frontotemporal Dementia (ALS-FTD) Continuum in Patients of Diverse Race, Ethnicity and Ancestry
Source: Genes (Basel). 2021 Dec 28;13(1):76. doi: 10.3390/genes13010076 (PMC8775163; doi:10.3390/genes13010076)
Supplement: Supplementary file 1 [file genes-13-00076-s001.zip › genes-1514337-supplementary.pdf]

Table S1. Variants Detected by Gene Panel Testing on C*9orf72* Negative Patients.

REA	Age at Testing	Variant	Interpretation
European Non-Finnish	71	TBK1 c.1341-1G>C(Splicing)	Likely Pathogenic
European Non-Finnish	61	SOD1 c.341T>C(p.Ile114Thr)	Pathogenic
European Non-Finnish	65	SOD1 c.14C>T(p.Ala5Val)	Pathogenic
European Non-Finnish	45	SOD1 c.341T>C(p.Ile114Thr)	Pathogenic
European Non-Finnish	47	SOD1 c.355G>C(p.Val119Leu)	Pathogenic
European Non-Finnish	53	SOD1 c.341T>C(p.Ile114Thr)	Pathogenic
European Non-Finnish	28	FUS c.1561C>G(p.Arg521Gly)	Pathogenic
European Non-Finnish	87	CDH13 c.778C>G(p.Leu260Val)	Uncertain
European Non-Finnish	89	ARHGEF28 c.490G>A(p.Glu164Lys)	Uncertain
European Non-Finnish	80	VAPB c.5C>T(p.Ala2Val)	Uncertain
European Non-Finnish	79	ARHGEF28 c.4024G>A(p.Gly1342Arg)	Uncertain
European Non-Finnish	78	PSEN2 c.208G>A(p.Gly70Arg)	Uncertain
European Non-Finnish	79	SETX c.7819G>A(p.Glu2607Lys)	Uncertain
European Non-Finnish	74	VCP c.374G>A(p.Gly125Asp)	Uncertain
European Non-Finnish	73	FUS c.1562G>A(p.Arg521His)	Pathogenic
European Non-Finnish	71	SOD1 c.413C>T(p.Thr138Ile)	Likely Pathogenic
European Non-Finnish	72	SOD1 c.193T>C(p.Phe65Leu)	Likely Pathogenic
European Non-Finnish	67	CHMP2B c.394A>G(p.Asn132Asp)	Uncertain
European Non-Finnish	69	GRN c.709-2A>G(Splicing)	Pathogenic
European Non-Finnish	69	SQSTM1 c.923C>T(p.Ala308Val)	Uncertain
European Non-Finnish	67	TARDBP c.941G>C(p.Gly314Ala)	Uncertain
European Non-Finnish	66	UBQLN2 c.1174A>G(p.Met392Val)	Uncertain
European Non-Finnish	67	ARHGEF28 c.133A>G(p.Met45Val)	Uncertain
European Non-Finnish	67	VCP c.79A>G(p.Ile27Val)	Uncertain
European Non-Finnish	67	CDH13 c.1894A>G(p.Ile632Val)	Uncertain
European Non-Finnish	67	SETX c.3029G>A(p.Arg1010His)	Uncertain
European Non-Finnish	64	GRN c.1664G>A(p.Arg555Gln)	Uncertain
European Non-Finnish	61	FUS c.676G>A(p.Gly226Ser)	Uncertain
European Non-Finnish	65	GRN c.328C>T(p.Arg110*)	Pathogenic
European Non-Finnish	62	GRN c.1224C>A(p.Tyr408*)	Likely Pathogenic
European Non-Finnish	60	VAPB c.112C>T(p.Arg38*)	Likely Pathogenic
European Non-Finnish	60	PSEN1 c.803T>C(p.Leu268Pro)	Uncertain
European Non-Finnish	58	GRN c.1402C>T(p.Gln468*)	Pathogenic
European Non-Finnish	60	SOD1 c.435G>C(p.Leu145Phe)	Pathogenic
European Non-Finnish	57	VCP c.277C>T(p.Arg93Cys)	Likely Pathogenic
European Non-Finnish	60	GRN c.970G>A(p.Ala324Thr)	Uncertain
European Non-Finnish	59	TBK1 c.1566C>G(p.Ile522Met)	Uncertain

European Non-Finnish	56	FUS c.188A>G(p.Asn63Ser)	Uncertain
European Non-Finnish	56	SQSTM1 c.663G>A(p.Thr221Thr)	Uncertain
European Non-Finnish	55	SQSTM1 c.1129C>G(p.Leu377Val)	Uncertain
European Non-Finnish	51	PSEN2 c.619A>G(p.Thr207Ala)	Uncertain
European Non-Finnish	48	PSEN1 c.488A>G(p.His163Arg)	Pathogenic
European Non-Finnish	45	ANG c.379G>A(p.Val127Ile)	Uncertain
European Non-Finnish	42	SOD1 c.139C>G(p.His47Asp)	Likely Pathogenic
European Non-Finnish	43	SOD1 c.14C>T(p.Ala5Val)	Pathogenic
European Non-Finnish	39	TREM2 c.313delG(p.Ala105Argfs*84)	Pathogenic
European Non-Finnish	34	SOD1 c.20G>T(p.Cys7Phe)	Pathogenic
European Non-Finnish	26	FUS c.1574C>G(p.Pro525Arg)	Uncertain
European Non-Finnish	84	TBK1 c.871A>G(p.Lys291Glu)	Uncertain
European Non-Finnish	78	SOD1 c.272A>C(p.Asp91Ala)	Likely Pathogenic
European Non-Finnish	71	FUS c.681_686del6(p.Gly230_Gly231del)	Suspected Pathogenic
European Non-Finnish	67	TBK1 c.65dupA(p.Asn22Lysfs*8)	Likely Pathogenic
European Non-Finnish	67	SOD1 c.272A>C(p.Asp91Ala)	Likely Pathogenic
European Non-Finnish	66	SOD1 c.435G>C(p.Leu145Phe)	Pathogenic
European Non-Finnish	65	SOD1 c.14C>T(p.Ala5Val)	Pathogenic
European Non-Finnish	64	TBK1 c.358+3A>G(Intronic)	Uncertain
European Non-Finnish	45	VCP c.572G>A(p.Arg191Gln)	Pathogenic
European Non-Finnish	40	SOD1 c.115C>G(p.Leu39Val)	Likely Pathogenic
European Non-Finnish	37	SOD1 c.305A>G(p.Asp102Gly)	Pathogenic
European Non-Finnish	65	PSEN1 c.337C>T(p.Leu113Leu)	Uncertain
European Non-Finnish	55	PSEN1 c.488A>G(p.His163Arg)	Pathogenic
European Non-Finnish	73	FUS c.*64T>G(Post-Coding)	Uncertain
European Non-Finnish	68	ARHGEF28 c.250A>G(p.Met84Val)	Uncertain
European Non-Finnish	57	SOD1 c.403A>C(p.Ser135Arg)	Likely Pathogenic
European Non-Finnish	86	SETX c.472T>G(p.Leu158Val)	Uncertain
European Non-Finnish	76	ARHGEF28 c.1717C>T(p.Arg573Cys)	Uncertain
European Non-Finnish	70	MAPT c.1822G>A(p.Gly608Arg)	Uncertain
European Non-Finnish	65	MAPT c.2014G>A(p.Val672Met)	Pathogenic
European Non-Finnish	65	ARHGEF28 c.4992G>A(p.Pro1664Pro)	Uncertain
European Non-Finnish	61	CDH13 c.1240G>C(p.Glu414Gln)	Uncertain
European Non-Finnish	62	MAPT c.1920+29G>A(Intronic)	Uncertain
European Non-Finnish	63	ANXA11 c.858+1G>A(Splicing)	Uncertain
European Non-Finnish	61	SOD1 c.169+52_169+58del(Intronic)	Uncertain
European Non-Finnish	57	MAPT c.1907C>T(p.Pro636Leu)	Pathogenic
European Non-Finnish	56	ANG c.122A>T(p.Lys41Ile)	Uncertain
European Non-Finnish	33	PSEN2 c.389C>T (p.Ser130Leu)	Uncertain
European Non-Finnish	77	SOD1 c.169+52_169+58del(Intronic)	Uncertain
European Non-Finnish	75	TARDBP c.1055A.G (p.Asn352Ser)	Pathogenic

European Non-Finnish	40	SOD1 c.413C>T(p.Thr138Ile)	Likely Pathogenic
European Non-Finnish	79	TARDBP c.881G>T(p.Gly294Val)	Likely Pathogenic
European Non-Finnish	54	PSEN1 c.488A>G(p.His163Arg)	Pathogenic
European Non-Finnish	20	CDH13 c.1609C>G(p.Leu537Val)	Uncertain
European Non-Finnish	57	PSEN1 c.525C>G(p.Phe175Leu)	Uncertain
European Finnish	58	SOD1 c.272A>C(p.Asp91Ala)	Likely Pathogenic
European Non-Finnish	68	FUS c.1181G>A(p.Arg394His)	Uncertain
Other	56	GRN c.396_397insG(p.Cys133Valfs*27)	Likely Pathogenic
Other	64	SOD1 c.412A>G(p.Thr138Ala)	Pathogenic
Other	70	MAPT c.1907C>T (p.Pro636Leu)	Pathogenic
Other	49	CHMP2B c.464A>G(p.Asp155Gly)	Uncertain
Other	66	TARDBP c.395T>C(p.Met132Thr)	Uncertain
Other	49	VCP c.1202A>G(p.Asn401Ser)	Uncertain
Other	53	SQSTM1 c.547C>T(p.Arg183Cys)	Uncertain
East Asian	66	TARDBP c.1135T>C(p.Ser379Pro)	Pathogenic
East Asian	51	ARHGEF28 c.5191C>T(p.Leu1731Phe)	Uncertain
South Asian	58	HNRNPA2B1 c.907T>C(p.Phe303Leu)	Uncertain
South Asian	39	ARHGEF28 c.5096C>T(p.Ala1699Val)	Uncertain
Mixed Non-European	65	PSEN1 c.617G>C(p.Gly206Ala)	Pathogenic
Mixed Non-European	84	SQSTM1 c.328C>T(p.Arg110Cys)	Uncertain
African	71	SOD1 c.124G>A(p.Gly42Ser)	Pathogenic
African	53	ARHGEF28 c.3533A>G(p.Asn1178Ser)	Uncertain
African	48	SOD1 c.301G>A(p.Glu101Lys)	Likely Pathogenic
African	47	SOD1 c.301G>A(p.Glu101Lys)	Likely Pathogenic
African	45	SOD1 c.420C>A(p.Asn140Lys)	Likely Pathogenic
Latino	67	MAPT c.1915G>A(p.Gly639Ser)	Likely Pathogenic
Latino	39	SOD1 c.68A>T(p.Gln23Leu)	Pathogenic
